# Sex Differences in Biochemical Analyses, Cardiometabolic Risk Factors and Their Correlation with CRP in Healthy Mexican Individuals

**DOI:** 10.3390/jpm14090904

**Published:** 2024-08-26

**Authors:** Aniel Jessica Leticia Brambila-Tapia, Alejandra Soledad González-Gómez, Laura Arely Carrillo-Delgadillo, Ana Míriam Saldaña-Cruz, Ingrid Patricia Dávalos-Rodríguez

**Affiliations:** 1 Departamento de Psicología Básica, Centro Universitario de Ciencias de la Salud (CUCS), Universidad de Guadalajara, Guadalajara 44340, Jalisco, Mexico; 2 Licenciatura en Nutrición, Centro Universitario de Ciencias de la Salud (CUCS), Universidad de Guadalajara, Guadalajara 44340, Jalisco, Mexico; alejandra.gonzalez5660@alumnos.udg.mx; 3 Licenciatura en Psicología, Centro Universitario de Ciencias de la Salud (CUCS), Universidad de Guadalajara, Guadalajara 44340, Jalisco, Mexico; laura.carrillo4391@alumnos.udg.mx; 4 Departamento de Fisiología, Centro Universitario de Ciencias de la Salud (CUCS), Universidad de Guadalajara, Guadalajara 44340, Jalisco, Mexico; ana.saldanac@academicos.udg.mx; 5 División de Genética, Centro de Investigación Biomédica de Occidente (CIBO), Instituto Mexicano del Seguro Social (IMSS), Guadalajara 44340, Jalisco, Mexico; 6 Departamento de Biología Molecular y Genómica, Centro Universitario de Ciencias de la Salud (CUCS), Universidad de Guadalajara, Guadalajara 44340, Jalisco, Mexico

**Keywords:** biochemical analyses, cardiometabolic risk factors, CRP, sex differences

## Abstract

Background: Few studies have been undertaken to detect the presence of cardiovascular risk factors (CRFs) in healthy populations (individuals auto-reported as healthy). These risk factors include high body mass index (BMI), high waist-to-hip ratio (WHR), high systolic and diastolic blood pressure (SBP, DBP), high uric acid and high Castelli’s risk index (CRI); this last is the ratio of total cholesterol to HDL cholesterol (TC/HDL-c). In addition, the correlations between CRFs and the biomarker C-reactive protein (CRP) has not been explored in each sex. Aim: Therefore, this study aimed to determine sex differences in the abnormalities in blood and urine analyses, including CRFs and their correlation with CPR in a non-representative sample of healthy Mexican individuals. Results: A total of 238 subjects were included, 123 (51.7%) of whom were women. The main blood alterations detected were high serum lipids, including high total cholesterol, LDL-cholesterol, triglycerides, and the CRI, which were higher in men than in women. The men’s samples had a higher frequency of hypertensives and pre-hypertensives than the women’s sample. The CRP showed positive significant correlations with the CRFs: BMI, WHR, SBP, DBP, uric acid, and the CRI, with a higher correlation for BMI and WHR, and most of these correlations were higher in women than in men. Additionally, all these factors showed a positive correlation among them. Conclusion: In conclusion, the main alterations observed in blood are related to cardiovascular risk and were reported with a higher frequency in men when compared with women. This finding can be related to the higher values of WHR in this sex; additionally, the inflammatory marker CRP was more correlated with the cardiometabolic risk factors in women than in men, which suggests a different relationship between inflammation and cardiometabolic risk factors in each sex.

## 1. Introduction

Preventive medicine is one of the most important areas of medical science, characterized by the prevention and early detection of chronic diseases. Among the diseases most commonly presented in the general population and which can be detected through biochemical analyses are anemia, urinary tract infections, chronic renal disease, diabetes, dyslipidemia, and hepatic inflammation; some of them are related to cardiovascular and other chronic diseases. For instance, an association between atherogenic indices and pre-eclampsia was documented [1]; in addition, an association between cardiometabolic risk factors and cardiorenal syndrome has also been reported [2]. On the other hand, the presence of cardiometabolic risk factors is highly frequent. In a previous study performed in Mexico, the prevalence of high cholesterol, triglycerides (TG), and obesity was reported in 40% of males and 36% of females with high cholesterol levels, 57.5% of males and 31.9% of females with high TG, and 48.1% of males and 37.2% of females with overweight/obesity [3].

Regarding the prevalence of other pathologies, which can be detected in general routine check-ups, it was reported that the detection of anemia in Mexican women of childbirth age with low economic status was observed in 34.3% of them [4]. Additionally, the presence of urinary tract infections (UTI) is rarely reported in healthy populations; however, a study aimed at detecting UTI in people with type 2 diabetes showed that these infections ranged from 17–31% [5,6], being more frequently reported in women than in men. Nevertheless, the report of laboratory values related to UTI, as well as the presence of anemia, high cholesterol, TG, glucose, and hepatic enzymes, among others, was not found in any healthy population including healthy Mexican individuals.

On the other hand, the correlation between the biomarker C-reactive protein (CRP) with well-known cardiovascular risk factors, including systolic blood pressure (SBP), body mass index (BMI), waist-to-hip ratio (WHR), uric acid and serum lipids, along with the presence of metabolic diseases and their influence in cardiovascular mortality, has been reported in some studies [7,8,9,10,11,12]. This relationship has been mainly explained by the influence of fatty tissue, which is accumulated in overweight and obesity, on inflammation, and high levels of total cholesterol (TC), low-density lipoprotein cholesterol (LDL-c), TG, and low levels of high-density lipoprotein cholesterol (HDL-c) [13,14], which contributes to the development of most cardiometabolic diseases [14]. It has also been shown that the atherogenic index in plasma (AIP) (_Log10_ TG/HDL-c) is positively related to CRFs including insulin resistance and waist circumference [15]. In addition to the AIP, other atherogenic indexes, including Castelli’s risk index I (CRI-I, TC/HDL-c) and II (LDL-c/HDL-c) have been associated with the presence of coronary artery disease [16], while the AIP has also been associated with the development of it [17]. However, the correlation between the CRP and CRFs, including SBP, BMI, WHR, uric acid, AIP, and CRI-I, has not been reported in a relatively healthy population (without self-reported chronic conditions) and separated by sex. This study could shed light with respect to the influence of each of these risk factors among them and in each sex, leading to the potential development of strategic preventive interventions adjusted by sex. 

Therefore, the objectives of this study were (a) to report the biochemical abnormalities in a wide range of blood and urine parameters, along with anthropometric and blood pressure (BP) measurements in a sample of healthy adult Mexican individuals, and to perform comparisons by sex; and (b) to determine the correlation between the inflammatory biomarker CRP and blood pressure and anthropometric and biochemical factors related to cardiovascular risk (atherogenic indices and uric acid) in a sample of healthy adult Mexican population separated by sex. In this sense, the hypotheses of our study are as follows: (a) there are sex differences in blood and urine abnormalities in a sample of Mexican individuals auto-reported as healthy and (b) the correlation between CRP and CRFs are different in each sex.

## 2. Subjects and Methods

### 2.1. Ethical Considerations and Study Population

The study was conducted according to the guidelines of the Declaration of Helsinki and was approved by the ethical committee of the Health Sciences University Center, with the registration number 19–21; the study was approved on 14 October 2019. All the participants signed an informed consent. All the participants were adult Mexican individuals, who were self-reported as healthy and lived in the area surrounding the city of Guadalajara, in the state of Jalisco, México.

### 2.2. Subjects

The selection criteria of the study were as follows: (a) to be older than 18 years old, (b) to have no have chronic or acute diseases, known by the subject (self-reported), and therefore not to be consuming any kind of medication, (c) subjects who were not consuming illegal drugs, (d) subjects who were not consuming hormonal products to increase muscular mass, (e) who were not pregnant, (f) subjects who were not genetically related to another participant of the study (i.e., brothers, cousins), and (g) subjects who preferably did not smoke. The elimination criterium was the absence of measurement of any variable.

#### 2.2.1. Study Design

Observational, cross-sectional study. This study consists of the measurement of several variables at the same point in time, which permits the establishment of possible relationships between them.

#### 2.2.2. Procedures

The invitation was conducted by an announcement distributed via social networks (WhatsApp and Facebook version 2022). Additionally, we invited university students personally in their classrooms; the distribution of the ad via these networks was performed over a period of 4 months, which was the time that the study lasted. The research team verified that all the subjects met the inclusion criteria, and if they accepted to participate, were cited in a computer room of the University of Guadalajara, where they signed an informed consent and filled out an electronic questionnaire (in Google Forms) that included personal variables. After filling out the electronic questionnaire, the anthropometric variables BMI and WHR were obtained. High BMI values (BMI > 25), were classified according to the standard classification, while the WHR was classified as high (men > 0.90, women > 0.86) according to the desirable values for the Mexican population [18]. Additionally, the systolic blood pressure (SBP) and diastolic blood pressure (DBP) were measured with an upper arm BP monitor brand Omron (model Hem-7320) in the left arm; we verified that the blood pressure values obtained with this device were replicated by the classic blood pressure cuff in the first and last participants of the study.

The blood and urine samples were obtained by personnel who worked for a certified laboratory. After the samples were obtained, they were transported to a biochemical laboratory, where the biochemical analyses were performed.

### 2.3. Personal Variables

The personal and sociodemographic variables included were as follows: sex; age; schooling; whether they had a job; whether they had a romantic partner; whether they had children; socioeconomic level; daily free hours; daily hours of physical activity; monthly extra money, which was measured with 5 categories, from nothing to more than USD 180; and alcohol and smoking consumption frequency, measured with 5 categories, from never to 4 or more times in the week.

### 2.4. Biochemical Variables Measurement

Analysis of biochemical variables was performed in a private and certificated laboratory (with EMMA and Joint Commission international certifications). Blood samples were obtained from all participants to quantify the following biochemical parameters: (1) complete blood count test (including hemoglobin, hematocrit, platelets, leucocytes, and their subpopulations); (2) complete lipid profile test (including total cholesterol, high-density lipoprotein (HDL), low-density lipoprotein (LDL), and TG). With these parameters, we obtained the CRI-I (ratio of TC to HDL cholesterol, TC/HDL-c) and the AIP (obtained by the log10 of the ratio of TG to HDL, _Log10_ TG/HDL-c); this index was classified as low risk if <0.11, intermediate risk from 0.11–0.21 and high risk if >0.21 [15]; (3) liver function test (alanine aminotransferase (ALT), aspartate aminotransferase (AST), gamma-glutamyl transferase (GGT), lactate dehydrogenase (LDH) enzymes, and alkaline phosphatase (ALP)); (4) blood chemistry (including uric acid, glucose, urea, creatinine, and blood urea nitrogen (BUN)); (5) serum electrolytes (calcium, phosphorus, magnesium, iron, sodium, potassium and chloride); (6) pancreatic enzymes (amylase and lipase), high-sensitivity CRP (measured with ELISA technique); and (7) general urine test, including leukocyte esterase, nitrites, erythrocytes and leukocytes.

The measurements were performed with electrical impedance for the blood count tests, colorimetry for all biochemical tests, and cytometry methods for urine tests. All the out-of-range values detected in the laboratory tests were double-checked (performed twice) to verify them.

### 2.5. Statistical Analysis

To describe the continuous variables, we used mean and standard deviations when the distribution was parametric and median and ranges when it was non-parametric. To describe categorical variables, we used frequencies and percentages. To compare sociodemographic variables between sexes, we used a chi-squared test for qualitative variables and Student’s *t*-test or the Mann–Whitney U test for quantitative ones (depending if the distribution was parametric or non-parametric, respectively). To compare categories of high and low values of each biochemical and anthropometric parameter, we used chi-squared and Fisher’s exact tests. To determine the correlation of the PCR values with the cardiovascular risk factors, we used the Pearson and Spearman correlation tests, depending on whether the distribution of the data was parametric or non-parametric, respectively. All analyses were performed with the software SPSS v.25, and a *p*-value < 0.05 was considered significant.

## 3. Results

A total of 238 participants were included, from whom 123 (51.7%) were women; the median was 24 and the age range was 18–69 years old. The personal and sociodemographic variables of participants in each sex are shown in Table 1, where we observe that both sexes were comparable in all variables except for daily free hours, which were higher in men than in women.

In relation to the descriptive values of the analyzed variables, we observed that the biochemical variables with the highest percentage of high abnormal values in men were LDL-c, TC, TG, CRI-I, AIP, calcium, hemoglobin, uric acid, LDH, and ALT enzymes in blood tests (Table 2).

In women, we observed that the variables with the highest percentages of high abnormal values in blood tests were as follows: LDL-c, TC, LDH and ALT enzymes, calcium and amylase pancreatic enzyme, while in urine tests, most variables, including leukocytes in urine, erythrocytes, and leukocyte esterase, showed a high level of abnormally high values. Concerning the abnormally low values in women, we observed that HDL cholesterol and leukocytes were the variables with the highest frequencies (Table 2).

Concerning anthropometric variables, we observed that BMI was the variable with the highest frequency of abnormally high values in both sexes, being higher in men, while WHR also showed a high frequency of abnormally high values in men (27.8%) (Table 3).

### 3.1. Comparison of Abnormal Values between Sexes

In Table 2, we show the high and low values of each studied blood, biochemical, and urine variable in each sex and their statistical comparison between sexes. There, we can observe that the men’s sample had a higher frequency of individuals with high TC, LDL-c, TG, CRI-I, and AIP than the women’s sample. In addition, in the parameters of uric acid, calcium, and iron levels, we observed that the men’s sample had a higher frequency of individuals with high frequencies of these variables when compared with the women’s sample. The women’s sample had a higher frequency of individuals with low iron levels and high levels of leukocyte esterase, leukocytes, nitrites, and erythrocytes in urine than the men’s sample. Finally, women also had higher levels of high CRP than men.

In Table 3, we show the differences in the anthropometric variables, as well as the differences of SBP and DBP between sexes. There, we can observe that the men’s sample showed a higher frequency of individuals with high values of WHR than the women’s sample. Concerning BP differences, the men’s sample had significantly higher levels of pre-hypertense and hypertense individuals in SBP and DBP when compared with the women’s sample (Table 3).

**Figure 1:**
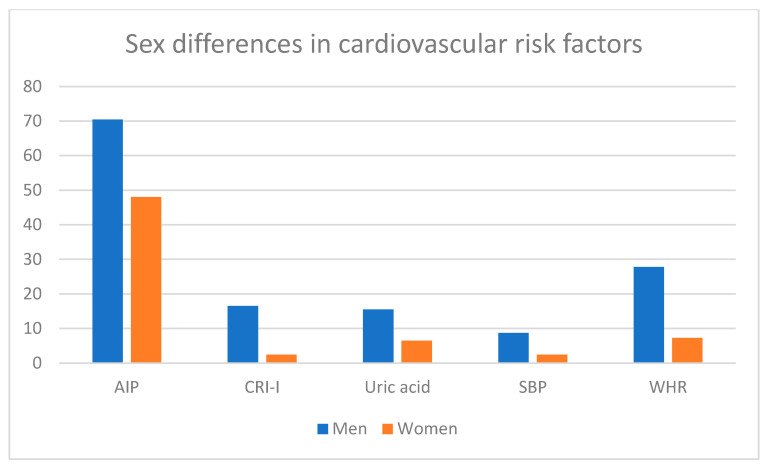
Percentages in the main cardiovascular risk factors in each sex.

**Figure 2:**
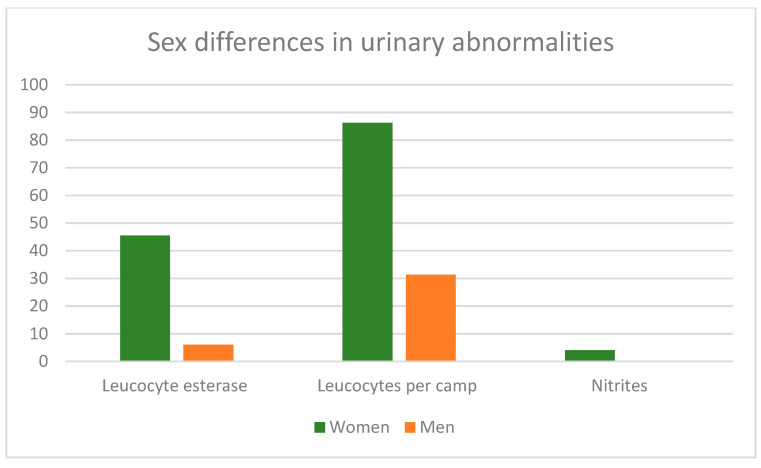
Percentages in the main urinary abnormalities in each sex.

The main sex differences in cardiovascular risk factors and urinary abnormalities are shown in Figure 1; Figure 2, where we can observe that men had a higher frequency of the main cardiovascular risk parameters (CRI-I, AIP, uric acid, SBP and WHR) than women (Table 1), while women showed a higher frequency of urinary abnormalities than men (Table 2).

### 3.2. Sex Comparisons in the Correlation between Cardiovascular Risk Factors and CRP

In the correlations between CRP and cardiovascular risk factors, we found that in women, CRP showed low to moderate correlation with all cardiovascular risk factors including BMI, WHR, CRI-I, AIP, SBP, SBP and uric acid, while in men, CRP showed low positive correlations with most of these factors, being moderate only for WHR. It is of interest that most of these correlations were higher in women than in men, except the anthropometric measures, where BMI showed a higher correlation with CRP in women, and WHR showed a higher correlation with CRP in men (Table 4; Table 5). Besides these differences, the correlations that showed more differences between sexes were the correlations between CRP and CRI-I and AIP, which were low in men and moderate in women, and the correlation between CRP and DBP, which was higher in women.

It was also observed that most cardiometabolic factors positively correlated amongst each other in both sexes, with WHR and BMI being the variables with the highest correlations with the rest of the factors.

## 4. Discussion

In the descriptive data, we observed that both sexes showed abnormally high levels of TC, LDL-c, and low levels of HDL-c; additionally, the men’s sample showed high levels of CRI-I, AIP, TG, and uric acid. For serum lipids, these observations coincide with a previous report performed on Mexican blood donors [19] (n = 1179), which showed a high percentage of individuals with high TC (48.7%), LDL-c (64.6%), TG (54%) and low HDL-c (52.4%). Although this study did not perform comparisons between sexes, we observed that their percentages of high levels of these serum lipids are higher than the frequency reported by our study; these discrepancies can be explained by the age of the studied participants, being in that study higher than in ours (44 years vs. 29 years); however, these data suggest that dyslipidemia is one of the main biochemical abnormalities found in routine laboratory analyses, coinciding with previous reports using a larger sample size of the Mexican population [19].

Additionally, when we compared these frequencies between sexes, we observed that men showed significantly higher levels of LDL-c, TG, CRI-I, AIP, and tendencies towards significance in the differences of TC, results that coincide with previous reports showing a higher frequency of dyslipidemia in men than in women in a Vietnamese population [20]. It is of interest that AIP was the CRF with the highest frequency in both sexes (Figure 1); this can be explained by the fact that AIP considers TG, while CRI-I does not. However, the high prevalence of individuals at high risk of AIP could indicate that Mexican individuals could be very prone to cardiovascular diseases. The higher frequency of dyslipidemia in men has been associated with higher abdominal adiposity in this sex [21,22,23,24], which was also confirmed in our results, where the frequency of high WHR was significantly higher in men than in women. This association can be explained by considering that the liver, located in the abdomen, is the organ responsible for lipid metabolism [25], and therefore, an increase in abdominal adiposity could increase LDL-c production.

The sex difference in WHR can also be related to the higher SBP and DPB in men when compared with women (Table 3), considering that a previous report by the research team found a significant moderate correlation between SBP and DBP with WHR [26]. In addition, the higher frequency of men with pre-hypertension and hypertension coincides with previous studies performed in Chinese and Spanish populations [27,28], a difference that can be explained by the biological effects of sex hormones on BP [29]. However, it has been shown that women show cardiovascular complications at lower levels of BP than men [30,31], questioning the precision of the thresholds established for each sex. Furthermore, it has been reported that hypertensive women are more frequent than hypertensive men in the elderly [32]. Nevertheless, a recent study showed higher age-adjusted mortality rates due to sleep apnea and health failure in elderly men when compared with elderly women in the United States [33]. All these data emphasize the need to perform more studies evaluating the effect of SBP and DBP variations adjusted by sex and age in different populations.

The high frequency of dyslipidemia in both sexes observed in our results is also in line with the high frequency of overweight/obesity observed in the participants, being slightly higher in men (46.1%) than in women (39.8%). When we compared these results with those reported in the literature, we observed that the frequencies in our results are similar to the frequency of overweight/obesity reported in a cohort of Mexican health workers (n = 9485), with 65.2% of males and 56.4% of females with overweight/obesity [3].

Concerning the frequency of individuals with high levels of uric acid, we observed that this was more than twice as frequent in men (16.5%) when compared with women (6.5%). The men/women ratio of high levels of uric acid was different to that reported by Robles-Rivera et al. 2022, in a study performed in 1423 Mexican health workers, where the authors found that 21.8% of men and 16.9% of women had high levels of uric acid (using the same cutoff values). In our study, these frequencies were lower, considering that we included healthy persons which additionally were of a lower age than in the Robles-Rivera report, whose participants had a median of age of 46 years old [34]. Concerning the high ALT values, a parameter related to NAFLD, we observed that the frequency in men (14.8%) was slightly lower than the frequency reported in a cohort of Mexican health workers (26.7%) [3]; in addition, in our results, this frequency was higher in men than in women (frequency of 9.8%), and this last was similar to that observed in the women’s population of this previous report (8.7%) [3].

Interestingly, for hemoglobin levels, we observed a low frequency of low hemoglobin values in women, although this was higher than the men’s frequency (4.1% vs. 0.0%, *p* = 0.06). These results differ from those reported in the Mexican population, where higher frequencies of anemia in women of different ages and socioeconomic status have been observed [4]; these discrepancies can be mainly explained by the socioeconomic status of the studied individuals, being in this population mainly “average/medium”. This category is related to sufficient income, which permits access to adequate nutrition. Furthermore, the higher frequency of the observed anemia in women than in men coincides with previous reports [35].

Finally, the abnormalities observed in the urine test (leukocyte esterase, leukocytes, nitrites, and erythrocytes) were clearly more frequent in women than in men. In the case of erythrocytes, this difference can be explained by the presence of menstruation and additionally by the presence of urinary tract infections, which have been reported with higher frequency in women than in men [36], and which are also related to the presence of leukocytes and leukocyte esterase [5]. These variables have shown high positive predictive values for detecting urinary tract infections in patients with type 2 diabetes [5]. Based on these parameters, around half of the included women would present a urinary tract infection.

Concerning the correlations between CRP and CRFs, we observed that women showed higher correlations between CRP with all these factors, which is an interesting finding not previously reported and suggests that inflammation could be more related to cardiometabolic risk in women than in men; in addition, this finding could be also related to the higher levels of CRP in women than in men previously observed by the research team [37] as well as in the present study.

Moreover, the fact that the risk factors most correlated with CRP were BMI for women and WHR for men could be related to the higher values of WHR observed in men than in women and coincides with the higher abdominal adiposity reported in this sex [18], as well as with the inflammatory effect of fatty tissue previously reported [13]. It is also of interest that the correlations between CRP with CRI-I and AIP were higher in women than in men (Table 4; Table 5). These results coincide with the sex differences in correlation values between BMI and CRP in the US population [7], in which higher correlation values were found in women (0.33) than in men (0.21). However, the correlations in this study were higher than those observed in the US report (see Table 4; Table 5), which suggests that serum lipid levels could be more related to inflammation in women. However, the causal direction of this relationship is unclear, and longitudinal studies should be performed in order to determine it. Additionally, when we compared the correlations between both atherogenic indices CRI-I and AIP with CRFs in each sex, we observed that AIP showed higher correlations with most CRFs than CRI-I in men, while in women, CRI-I showed higher correlations than AIP. These results suggest that AIP can be a better atherogenic index in men, while CRI-I can be better in women. Nevertheless, for both sexes, AIP showed higher correlations with CRP than CRI-I, which could be related to the fact that AIP has been reported as a better predictor of coronary artery disease than CRI-I and other atherogenic indices [16].

Finally, the positive correlations between most of these factors in both sexes, where the anthropometric measures showed the highest correlations, suggest that the accumulation of fatty tissue observed in overweight/obesity is the main risk factor that influences the other CRFs and also coincides with a report showing that abdominal obesity is associated with multimorbidity of cardiometabolic diseases [14]. This can be explained by the inflammatory pathway in the development of these diseases; however, the specific influence of each cardiometabolic risk factor in the development of cardiometabolic diseases remains to be determined in each sex.

The main limitations of the study are the small sample size and the inclusion of mainly young people, which along with the non-random sampling method means that the study is not representative of the Mexican population.

These limitations could bias the results by considering that the inclusion of larger sample sizes and older people could determine the main biochemical abnormalities present in the general healthy Mexican population and could detect more accurate correlations between the studied variables (CRFs and CRP). In this sense, when we compare the sex differences of serum lipids in the results obtained with studies with larger samples and with older individuals of the Mexican population [19], we observe that larger samples with older individuals show higher abnormalities in lipid levels with higher differences between sexes, with men having more than double the frequency of individuals with hypercholesterolemia and hypertriglyceridemia than women [19]. Concerning the frequency of overweight/obesity, we also observe that higher values of this variable were observed in larger samples and older individuals of the Mexican population [3], although sex differences remain similar to those found in our report. Additionally, high ALT values were more frequent in the men’s sample of larger samples of the Mexican population [3] than in our results, similar to the frequency of individuals with high levels of uric acid, which have been reported with a higher frequency in larger samples including older individuals of the Mexican population [34], with lower differences between sexes than those found in this report.

The influence of age in these abnormalities can be explained by the positive correlation between age and BMI and WHR, as observed in this study, and the fact that at older ages there are higher possibilities of oxidative stress and morbidity [38]. Therefore, it is expected that CRFs will be more present at older ages. In addition to age, other risk factors such as male sex, social disadvantage, and genetic predisposition have been associated with cardiovascular disease development [39,40], factors that could have influenced the results of this study.

An additional limitation of the study is that the bivariate correlations performed among the CRFs could be influenced by other factors; therefore, multivariate analyses with larger sample sizes would clarify these observations. However, despite these limitations, this study adds new and useful information not previously reported, including the analyses of many biochemical variables and their comparison between sexes as well as the correlation between the main CRFs and CRP in each sex.

In conclusion, the main biochemical abnormalities detected in blood tests were high serum lipids, which were higher in men than in women. In addition, high levels of uric acid, a variable also related to cardiovascular risk [41], were also detected with a higher frequency in men than in women. In addition, the main urinary alterations included the presence of erythrocytes, leukocytes, and leukocyte esterase, which were much more frequent in women than in men. We observed that the frequency of high values of WHR was significantly higher in men than in women. Concerning the correlations between CRFs and CRP, we observed that most of these factors showed a positive correlation with CRP in both sexes, being higher in women than in men; in addition, all the risk factors showed a positive correlation amongst themselves in both sexes, being the anthropometric measures BMI and WHR, the CRF that showed the strongest correlation with the rest of them. Therefore, the prevention of overweight and obesity, which has constantly increased in Mexico [42] and is projected to increase in occidental countries [43], is fundamental to preventing mortality rates associated with cardiometabolic diseases. Further larger studies with a wider variety of ages performed in Mexican and other populations will clarify these results.

## Figures and Tables

**Table 1:** Personal and sociodemographic variables of participants according to sex.

**Variable**	**Women (n = 123)**	**Men (n = 115)**	***p* Value**
Age, mean ± SD	29.34 ± 10.54	28.73 ± 11.70	0.336
With romantic partner, n (%)	70 (56.9)	72 (62.6)	0.428
With children, n (%)	38 (30.9)	22 (19.1)	0.052
With job, n (%)	74 (60.2)	67 (58.3)	0.793
Schooling, n (%)			0.768
Elementary school	1 (0.8)	1 (0.9)	
Secondary	5 (4.1)	5 (4.3)	
Preparatory	53 (43.1)	55 (47.9)	
University (Bachelor’s degree)	51 (41.5)	40 (34.8)	
Master’s degree	11 (8.9)	9 (7.8)	
Ph.D. degree	2 (1.6)	5 (4.3)	
Socioeconomic level, n (%)			0.144
Very low	0 (0.0)	2 (1.7)	
Low	17 (13.8)	17 (14.8)	
Average	105 (85.4)	91 (79.2)	
High	1 (0.8)	5 (4.3)	
Very high	0 (0.0)	0 (0.0)	
Monthly extra money, mean ± SD	2.82 ± 1.17	2.97 ± 1.28	0.341
Daily hours of physical activity, median (range)	1 (0–6.4)	1 (0–6.4)	0.942
Daily free hours, median (range)	4 (0–10)	4 (1–16)	0.021
Smoking frequency, median (range)	0 (0–4)	0 (0–4)	0.929
Alcohol consumption frequency, median (range)	1.47 ± 0.94	1.62 ± 0.99	0.411

*p* value obtained with chi-squared test, Student’s *t*-test, and Mann–Whitney U test. Monthly extra money: 5 categories, from nothing to more than USD 180. Smoking and alcohol consumption frequency were measured from 0–4 (never to more than 4 times in the week).

**Table 2:** Comparison of the percentages of high and low values of the studied biochemical variables between sexes.

Variable, Units º	High Values, n (%)		Low Values, n (%)		*p* Value (for High)	*p* Value (for Low)	Reference Values ª
	Women	Men	Women	Men			
**Sample Size**	**Women (N = 123)/Men (N = 115)**						
Hemoglobin, g/dL	2 (1.6)	25 (21.7)	5 (4.1)	0 (0.0)	<0.0001	0.06	W: 12.00–16.00 M: 14.00–17.00
Leukocytes, 10^3^/μL	4 (3.3)	5 (4.3)	11 (8.9)	18 (15.7)	0.74	0.16	5.00–10.00
Monocytes, 10^3^/μL	0 (0.0)	1 (0.9)	0 (0.0)	0 (0.0)	0.48	1.00	0.10–1.00
Lymphocytes, 10^3^/μL	0 (0.0)	1 (0.9)	1 (0.8)	0 (0.0)	0.48	1.00	1.00–4.20
Platelets, 10^3^/μL	4 (3.3)	2 (1.6)	0 (0.0)	0 (0.0)	0.68	1.00	141–400
**Serum lipids**							
Total cholesterol, mg/dL	19 (15.4)	29 (25.2)	-	-	0.07	-	≤200.00
Low-density lipoprotein (LDL), mg/dL	59 (48.0)	74 (64.3)	-	-	0.01	-	≤100.00
High-density lipoprotein (HDL), mg/dL	-	-	37 (30.1)	18 (15.7)	-	0.009 **	W > 45.00 M > 35.00
Triglycerides, mg/dL	15 (12.2)	34 (29.6)	-	-	0.001	-	≤150.00
Castelli’s risk index I (CRI-I)	3 (2.4)	19 (16.5)	-	-	<0.001	-	<5.00
Atherogenic index in plasma (AIP)	59 (48.0)	81 (70.4)	-	-	<0.001	-	<0.21
**Liver function tests**							
Aspartate aminotransferase (AST), U/L	7 (5.7)	8 (7.0)	-	-	0.79	-	W ≤ 32.00 M ≤ 40.00
Alanine aminotransferase (ALT), U/L	12 (9.8)	17 (14.8)	-	-	0.32	-	W ≤ 33.00 M ≤ 41.00
Gamma-glutamyl transferase (GGT), U/L	6 (4.9)	7 (6.1)	-	-	0.77	-	W ≤ 40.00 M ≤ 60.00
Alkaline phosphatase (ALP), U/L	8 (6.5)	12 (10.4)	1 (0.8)	0 (0.0)	0.35	1.00	W: 35.00–104.00 M: 40.00–129.00
Lactate dehydrogenase (LDH), U/L	34 (27.6)	38 (33.0)	8 (6.5)	4 (3.5)	0.39	0.37	W: 135.00–214.00 M: 135.00–225.00
**Blood chemistry**							
Glucose, g/dL	3 (2.4)	4 (3.5)	8 (6.5)	2 (1.7)	0.71	0.10	74.00–106.00
Urea, mg/dL	1 (0.8)	2 (1.7)	3 (2.4)	1 (0.9)	0.61	0.62	16.60–48.50
Creatinine, mg/dL	8 (6.5)	2 (1.7)	1 (0.8)	4 (3.5)	0.10	0.20	W: 0.50–0.90 M: 0.70–1.20
Uric acid, mg/dL	8 (6.5)	19 (16.5)	3 (2.4)	0 (0.0)	0.02	0.25	W: 2.40–5.70 M: 3.40–7.00
**Serum electrolytes**							
Calcium, mg/dL	14 (11.4)	37 (32.2)	0 (0.0)	0 (0.0)	<0.001	1.00	8.60–10.00
Sodium, mg/dL	4 (3.3)	1 (0.9)	5 (4.1)	2 (1.7)	0.37	0.44	136.00–145.00
Potassium, meq/L	8 (6.5)	6 (5.2)	0 (0.0)	0 (0.0)	0.78	1.00	3.50–5.10
Magnesium, mg/dL	0 (0.0)	0 (0.0)	0 (0.0)	0 (0.0)	1.00	1.00	1.60–2.60
Iron, μg/dL	0 (0.0)	5 (4.3)	7 (5.7)	0 (0.0)	0.025	0.014	33.00–193.00
Phosphorus, mg/dL	1 (0.8)	2 (1.7)	4 (3.3)	0 (0.0)	0.611	0.122	2.50–4.50
Chloride, meq/L	7 (5.7)	2 (1.7)	0 (0.0)	2 (1.7)	0.174	0.232	98.00–107.00
**Pancreatic enzymes**							
Amylase, U/L	13 (10.6)	9 (7.8)	3 (2.4)	0 (0.0)	0.509	0.248	28.00–100.00
Lipase, U/L	5 (4.1)	1 (0.9)	0 (0.0)	0 (0.0)	0.214	1.00	13.00–60.00
**C-reactive protein**	28 (22.8)	14 (12.2)	-	-	0.04	-	0.10–3.00
**General urine test**							
Leucocyte esterase (> 0)	56 (45.5)	7 (6.1)	-	-	<0.0001	-	0
Erythrocytes (≥ 1 per camp	73 (59.3)	39 (33.9)	-	-	<0.0001	-	0
Leucocytes (>1 per camp)	106 (86.2)	36 (31.3)	-	-	<0.0001	-	0
Nitrites (≥ 1 per camp)	5 (4.1)	0 (0.0)	-	-	0.06	-	0

*p* values obtained with Fisher’s exact test. º Units in which the variable was measured; however, all the values in the table correspond to the numbers and percentages of high and low values of each variable in each sex. ª References values according to the laboratory. M: Men, W: Women.

**Table 3:** Comparison of the percentages of high values of body anthropometric measures between sexes.

Variable	Women, n (%) Women = 123	Men, n (%) Men = 115	*p* Value
Body mass index (BMI) > 25.0	49 (39.8)	53 (46.1)	0.36
Waist-to-hip ratio (WHR), M > 0.90, W > 0.86	9 (7.3)	32 (27.8)	<0.0001
Systolic blood pressure (SBP)			<0.001
Normal ≤ 120 mmHg	113 (91.9)	57 (49.6)	
Pre-hypertension (121–139 mmHg)	7 (5.7)	48 (41.7)	
High ≥ 140 mmHg	3 (2.4)	10 (8.7)	
Diastolic blood pressure (DBP)			0.009
Normal ≤ 80 mmHg	95 (77.2)	68 (59.1)	
Pre-hypertension (81–89 mmHg)	21 (17.1)	31 (27.0)	
High ≥ 90 mmHg	7 (5.7)	16 (13.9)	

*p* value obtained with Fisher’s exact test, W: women, M: men.

**Table 4:** Correlations between CRP and cardiometabolic risk factors in women.

Variable	Age	BMI	WHR	SBP	DBP	Uric Acid	CRI-I	AIP
CRP	0.297 *	0.526 **	0.304 **	0.248 **	0.363 **	0.294 **	0.412 **	0.469 **
Age	-	0.384 **	0.443 **	0.294 **	0.268 *	0.116	0.396 **	0.375 **
BMI	-	-	0.485 **	0.324 **	0.302 **	0.145	0.587 **	0.515 **
WHR	-	-	-	0.215 *	0.193 *	0.214 *	0.427 **	0.378 **
SBP	-	-	-	-	0.722 **	0.236 **	0.141	0.082
DBP	-	-	-	-	-	0.252 **	0.113	0.114
Uric acid	-	-	-	-	-	-	0.202 *	0.239 **
CRI-I	-	-	-	-	-	-	-	0.810 **

CRP: C-reactive protein, BMI: body mass index, WHR: waist-to-hip ratio, SBP: systolic blood pressure, DBP: diastolic blood pressure, CRI-I: Castelli’s risk index I, AIP: atherogenic index in plasma. *p* values obtained with Pearson and Spearman correlation tests. * *p* < 0.05, ** *p* < 0.01.

**Table 5:** Correlations between CRP and cardiometabolic risk factors in men.

Variable	Age	BMI	WHR	SBP	DBP	Uric Acid	CRI-I	AIP
CRP	0.184	0.393 **	0.402 **	0.116	0.190 *	0.218 *	0.277 **	0.287 **
Age	-	0.352 **	0.680 **	0.095	0.349 **	0.069	0.455 **	0.512 **
BMI	-	-	0.595 **	0.324 **	0.274 **	0.275 *	0.376 **	0.368 **
WHR	-	-	-	0.181	0.336 **	0.353 **	0.573 **	0.599 **
SBP	-	-	-	-	0.656 **	0.19	0.174	0.123
DBP	-	-	-	-	-	0.260 **	0.193 *	0.301 **
Uric acid	-	-	-	-	-	-	0.099	0.234 **
CRI-I	-	-	-	-	-	-	-	0.815 **

CRP: C-reactive protein, BMI: body mass index, WHR: waist-to-hip ratio, SBP: systolic blood pressure, DBP: diastolic blood pressure, CRI-I: Castelli’s risk index I, AIP: atherogenic index in plasma. *p* values obtained with Pearson and Spearman correlation tests. * *p* < 0.05, ** *p* < 0.01.

## Data Availability

The datasets presented in this article are not readily available because there is personal information in the database. Requests to access the datasets should be directed to the corresponding authors.
